# Supplementary material for: Oncolytic Herpes Simplex Virus Type 1 Induces Immunogenic Cell Death Resulting in Maturation of BDCA-1+ Myeloid Dendritic Cells
Source: Int J Mol Sci. 2022 Apr 27;23(9):4865. doi: 10.3390/ijms23094865 (PMC9103433; doi:10.3390/ijms23094865)
Supplement: Supplementary file 1 [file ijms-23-04865-s001.zip › ijms-1684849-supplementary.pdf]

Figure S1

DX **STS** **SFZ**

(a) 624-mel

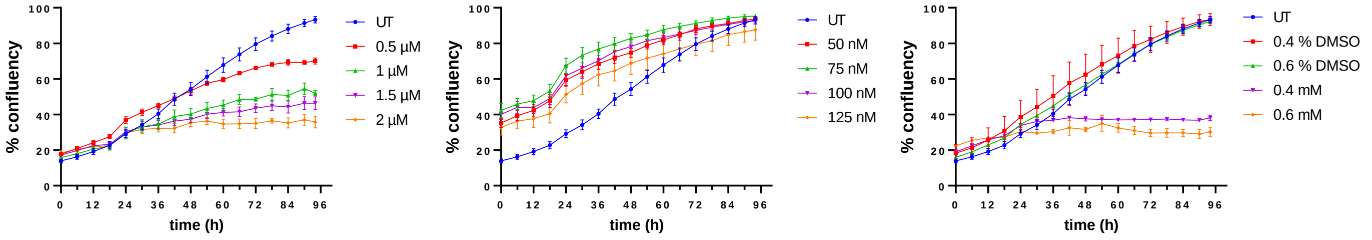

(b) 888-mel

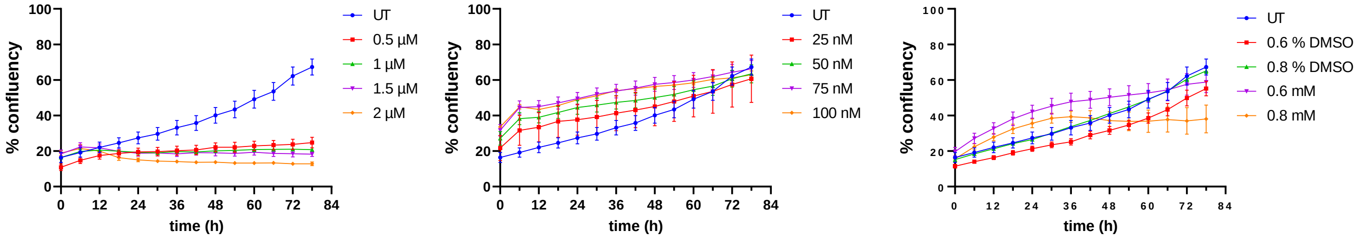

(c) 938-mel

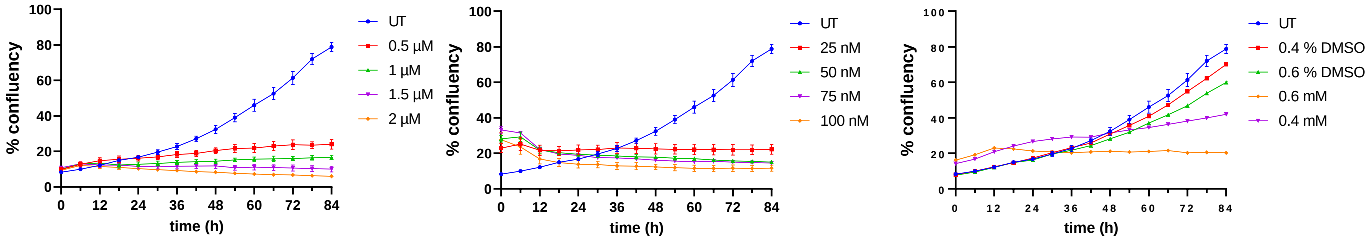

(d) MZ2

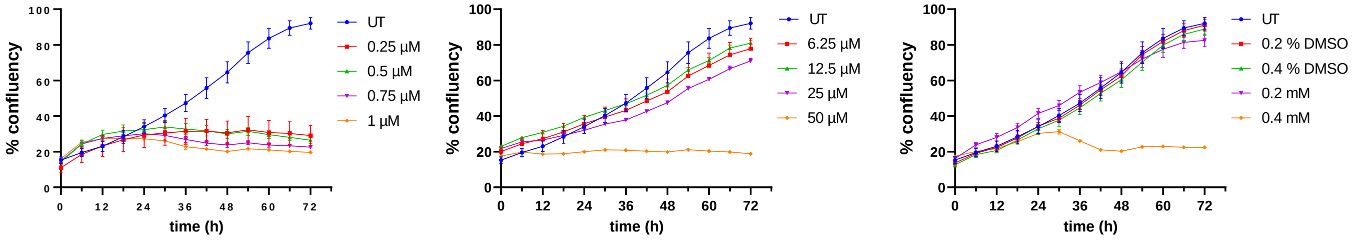

Figure S1: Titration of CDI agents DX, STS and SFZ on several melanoma cell lines. (a) 624-mel (7 500 cells/well), (b) 888-mel (10 000 cells/well), (c) 938-mel (7500 cells/well) and (d) MZ2 (7 500 cells / well) cells were seeded in a 96-well flat bottom plate and incubated overnight. The next day, cells were treated with DX, STS or SFZ at indicated concentrations and cell confluency was monitored for up to 96h using the IncuCyte® analysis system. The experiment was conducted in a single repeat with three technical replicats. Data was analysed using the IncuCyte® ZOOM software. Error bars display SEM. Abbreviations: CDI: cell death inducing ; DMSO: dimethyl sulfoxide ; DX: doxorubicin ; SEM: standard error of the mean ; STS: staurosporine ; SFZ: sulfasalazine ; UT: untreated.

Figure S2

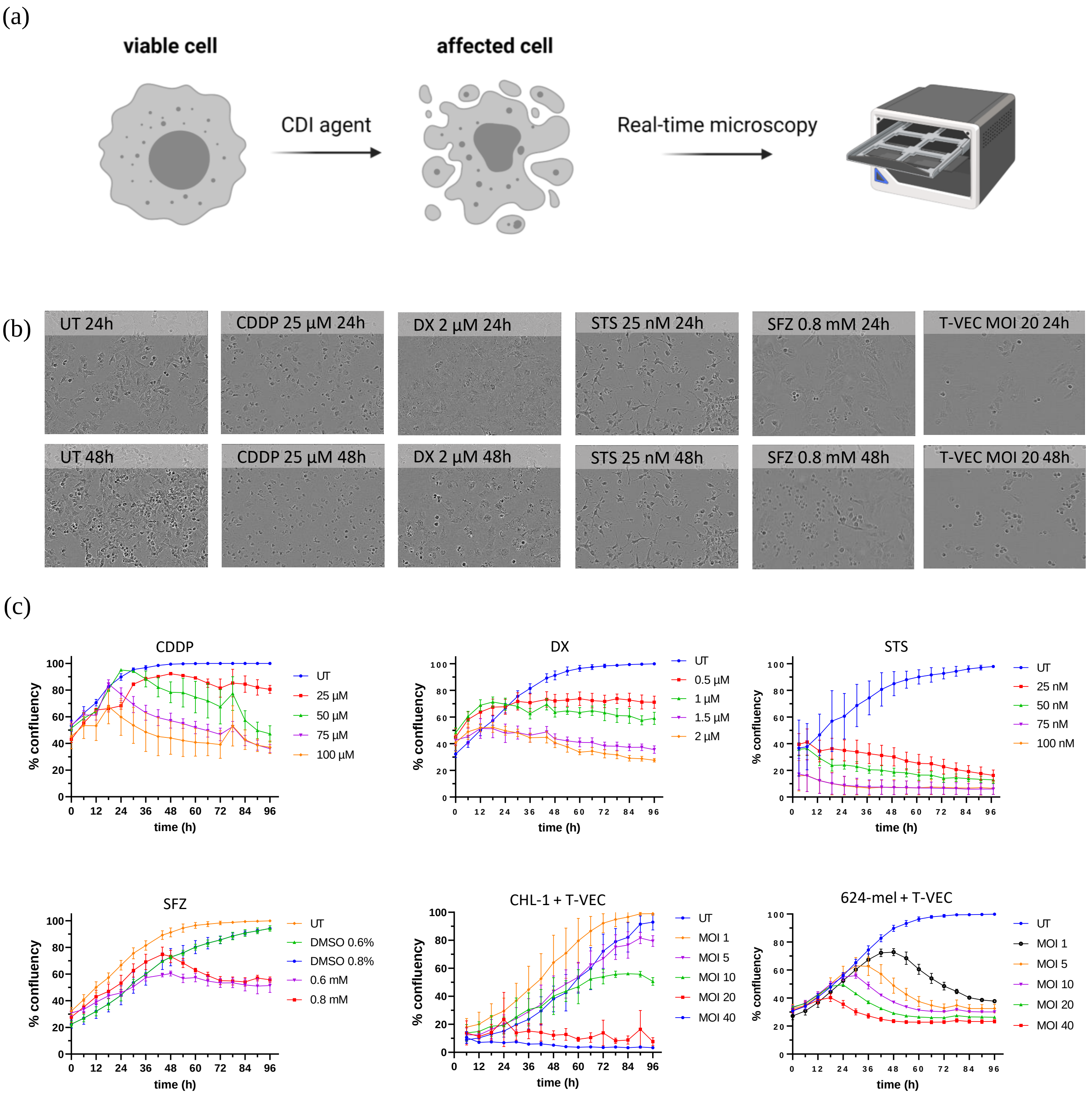

Figure S2: Titration of CDI agents CDDP, DX, STS, SFZ and T-VEC on melanoma cells using IncuCyte® ZOOM analysis system. (a) CHL-1 cells were seeded in a 96-well flat bottom plate and incubated overnight. The next day, cells were treated with CDDP, DX, STS, SFZ or T-VEC at indicated concentrations / MOI and cell confluency was monitored for up to 96h using the IncuCyte® analysis system. The experiment was conducted in single repeat with three technical replicates. Data was analyzed using the IncuCyte® ZOOM software. (b) Representative microscopic pictures acquire with the IncuCyte® analysis system at 24h and 48h of selected conditions are shown. (c) Graphs showing proliferation curves of CHL-1 cells (CDDP, DX, STS, SFZ or T-VEC) or 624-mel cells (T-VEC) in the course of time. Error bars display SEM. Abbreviations: CDDP: cisplatin ; CDI: cell death inducing ; DX: doxorubicin ; MOI: multiplicity of infection ; SEM: standard error of the mean ; STS: staurosporine ; SFZ: sulfasalazine ; T-VEC: Talimogene laherparepvec ; UT: untreated.

Figure S3

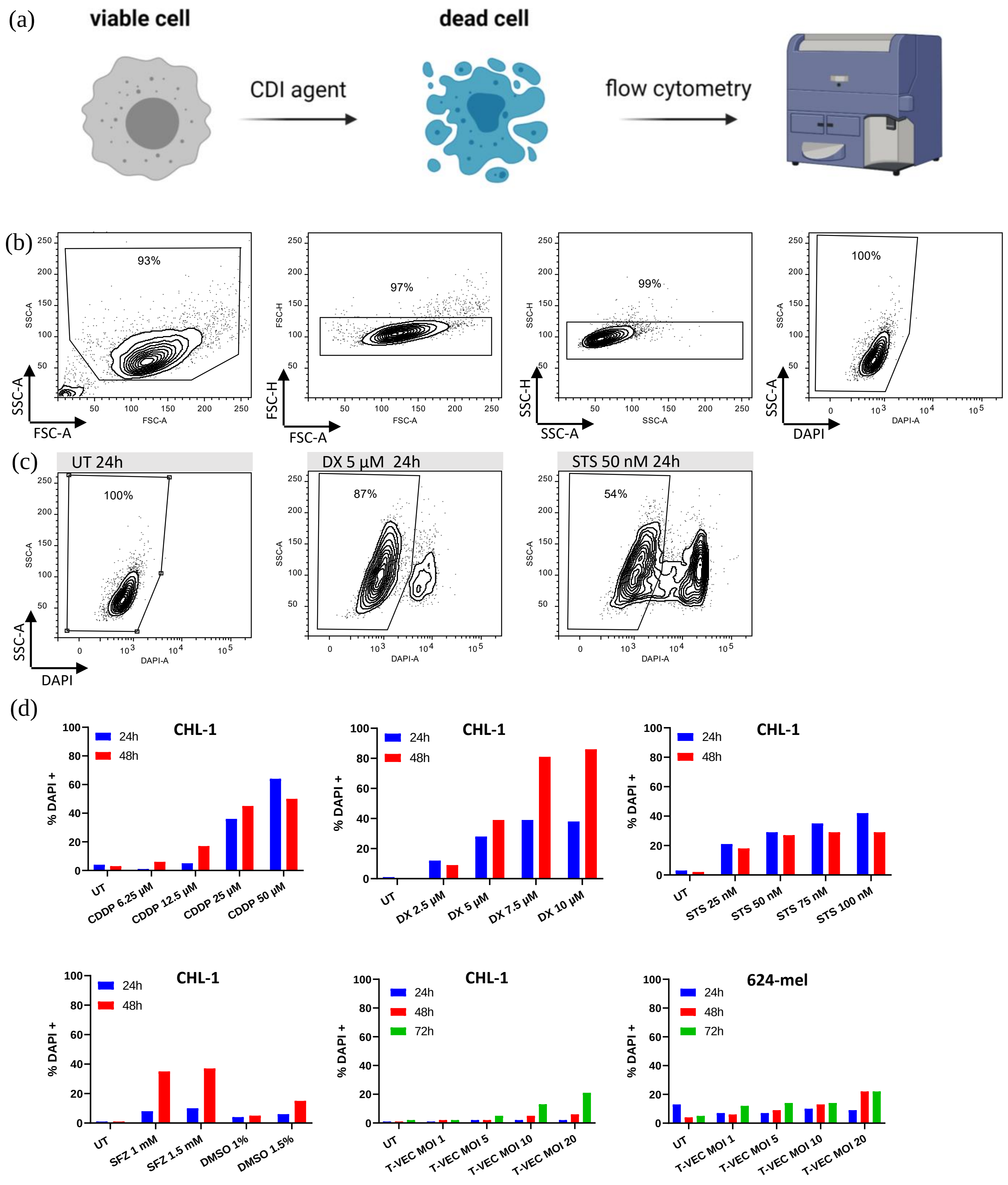

Figure S3: Flow cytometry based titration of CDI agents CDDP, DX, STS, SFZ or T-VEC on CHL-1. (a) CHL-1 cells were seeded in a 12-well plate and incubated overnight. The next day, cells were treated with CDDP, DX, STS, SFZ or T-VEC at indicated concentrations for up to 48h. Cells were harvested, DAPI stained and analyzed using flow cytometry. (b) Representative contour plots illustrating the FSC/SSC gating strategy for single cells. (c) Within single cells, cells were gated on in DAPI negative cells. (d) Bar graphs from single repeat experiment showing percentage DAPI^{+} cells. Abbreviations: CDI: cell death inducing ; CDDP: cisplatin ; DMSO: dimethyl sulfoxide ; DX: doxorubicin ; MOI: multiplicity of infection ; SFZ: sulfasalzine ; STS: staurosporine ; T-VEC: Talimogene laherparepvec ; UT: untreated.

<u>Figure S4</u>

(a)

(b)

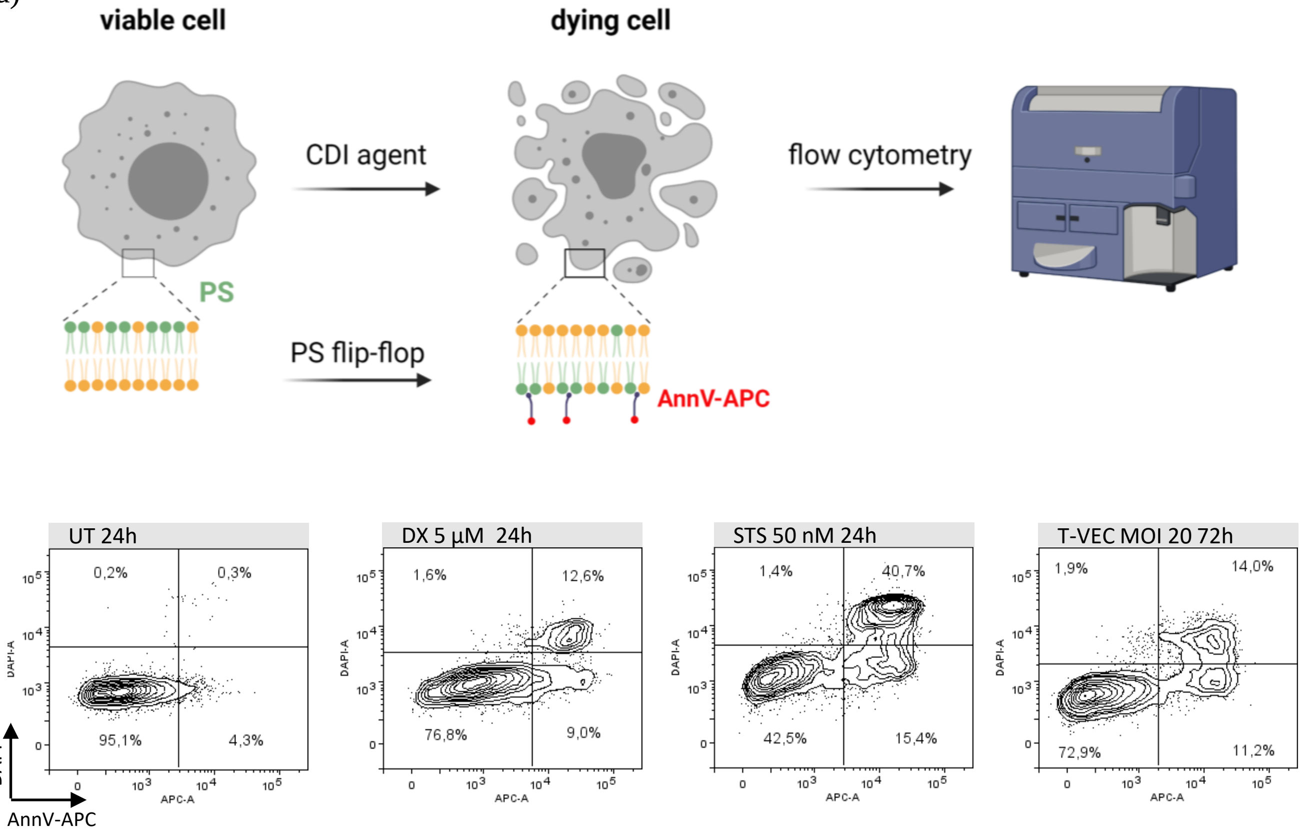

Figure S4: Assay for the detection of PS exposing cells. (a) CHL-1 cells were seeded a 12-well plate and incubated overnight. The next day, cells were treated with CDI agents. After treatment, cells were collected, stained with Annexin V / DAPI and analyzed by means of flow cytometry. (b) Representative contour plots showing gating strategy to identify Annexin V positive cells within the DAPI negative population. Abbreviations: AnnV: Annexin V ; CDI: cell death inducing ; CDDP: cisplatin ; DX: doxorubicin ; MOI: multiplicity of infection ; PS: phosphatidylserine ; STS: staurosporine ; T-VEC: Talimogene laherparepvec ; UT: untreated.

Figure S5

(a)

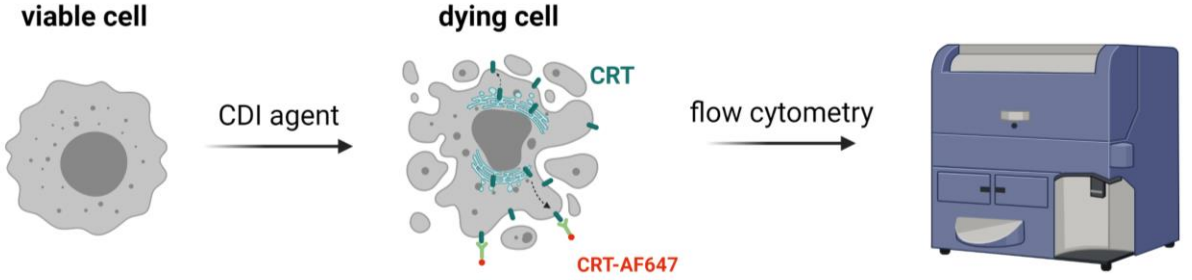

(b)

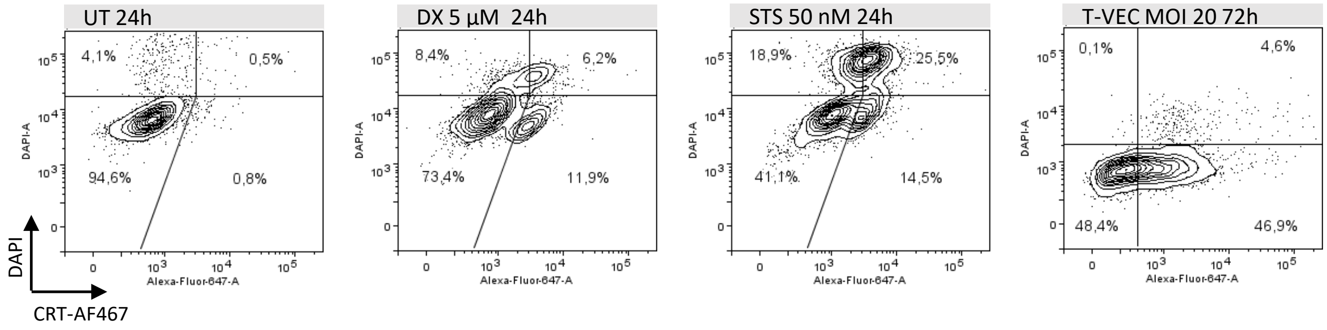

Figure S5: Assay for the detection of surface CRT exposing cells. (a) CHL-1 cells were seeded a 12-well plate and incubated overnight. The next day, cells were treated with CDI agents. After treatment, cells were collected, stained with anti-CRT antibody / DAPI and analyzed using flow cytometry. (b) Representative contour plots showing gating strategy to identify CRT positive cells within the DAPI negative population. Abbreviations: CDI: cell death inducing ; CRT: calreticulin ; DX: doxorubicin ; MOI: multiplicity of infection ; STS: staurosporine; T-VEC: Talimogene laherparepvec ; UT: untreated.

Figure S6

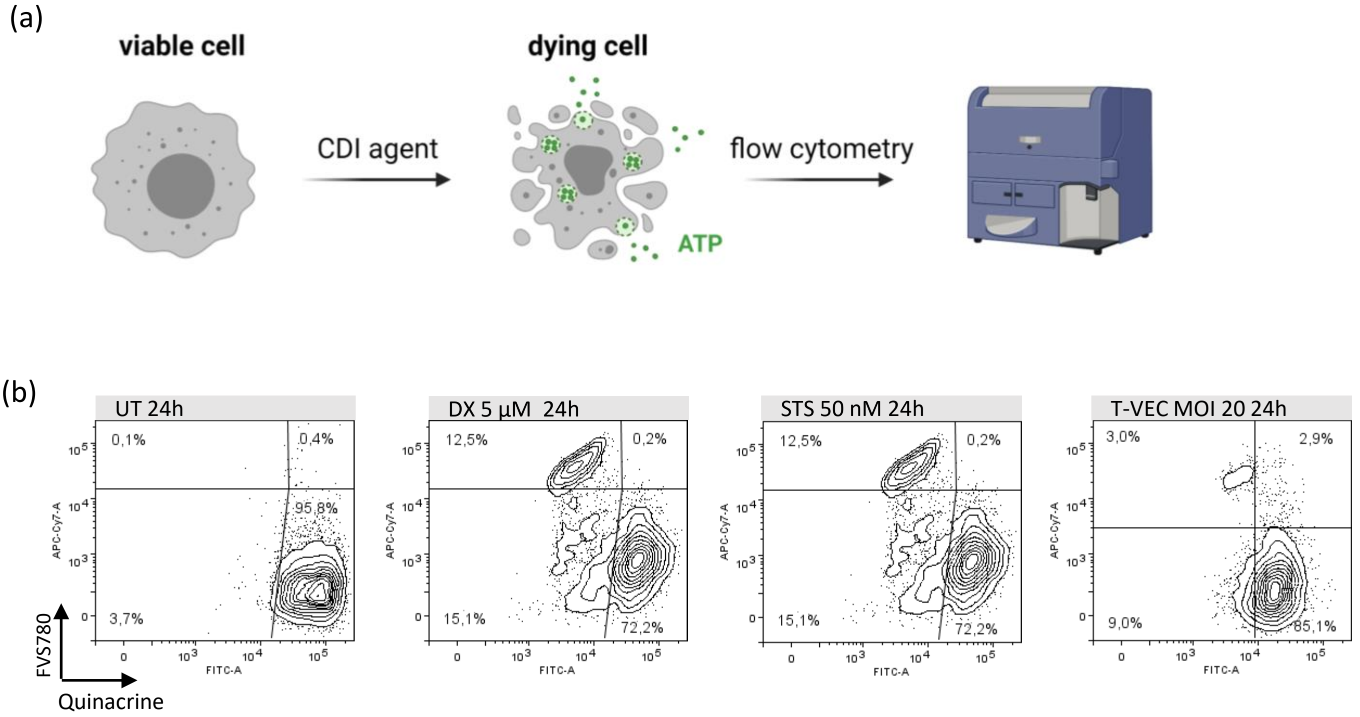

Figure S6: Assay for the detection of ATP releasing cells. (a) CHL-1 cells were seeded a 12-well plate and incubated overnight. The next day, cells were treated with CDI agents. After treatment, cells were collected, stained with FVS780 / quinacrine and analyzed by means of flow cytometry (b) Representative contour plots, showing gating strategy to identify ATP releaseing cells within the FVS780 negative population. Abbreviations: CDI: cell death inducing ; DX: doxorubicin ; MOI: multiplicity of infection ; STS: staurosporine ; T-VEC: Talimogene laherparepvec ; UT: untreated.

Figure S7

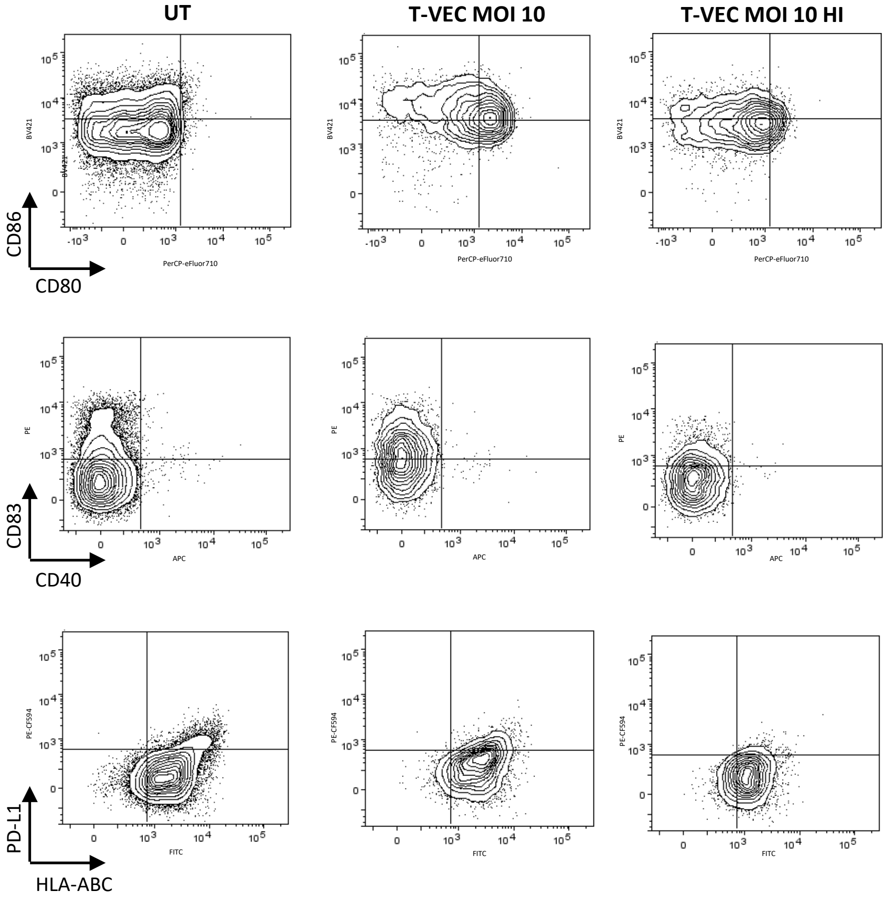

Figure S7: Representative plots concerning the maturation of BDCA-1^{+} myDCs after exposure with (in)activated T-VEC. Representative contour plots, showing the expression of CD86, CD80, CD83, CD40 and HLA-ABC on BDCA-1^{+} DCs after treatment with active or heat-inactivated T-VEC. As a negative control, cells were left untreated. Abbreviations: myDCs: myeloid dendritic cells ; HI: heat-inactivated ; T-VEC: Talimogene laherparepvec ; UT: untreated.

<u>Figure S8</u>

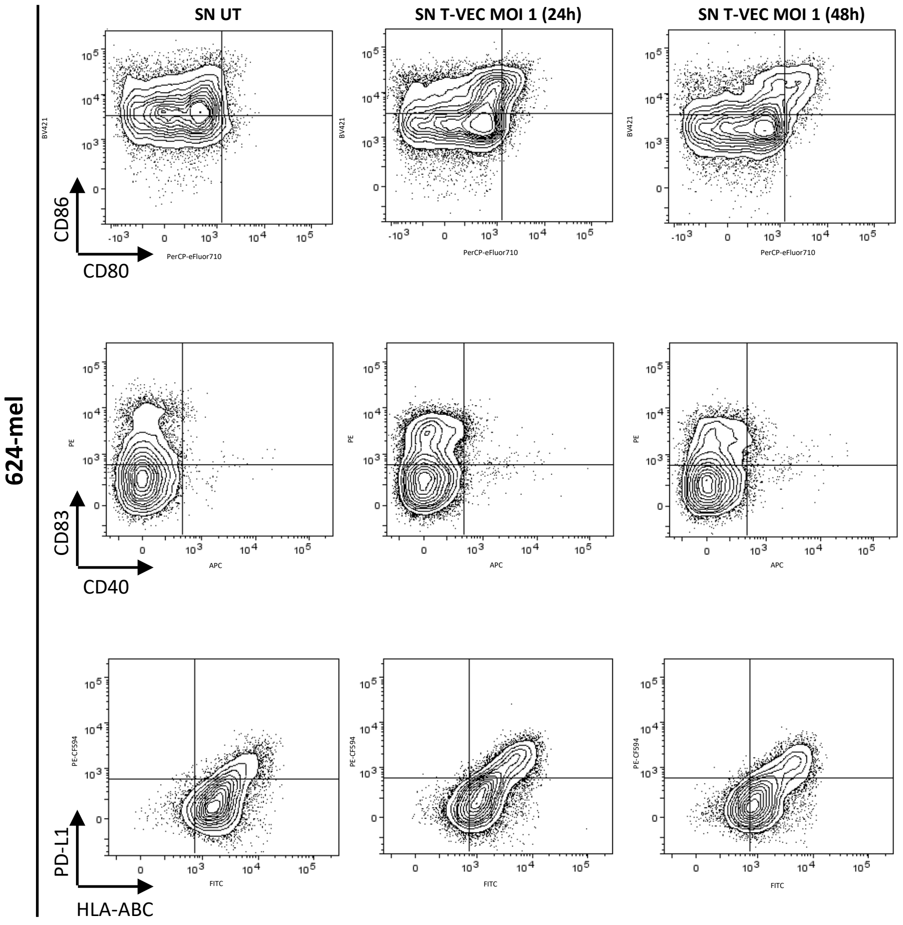

Figure S8: Representative plots concerning the maturation of BDCA-1$^+$ myDCs after treatment with conditioned medium. Representative contour plots show the expression of CD86, CD80, CD83, CD40 and HLA-ABC on BDCA-1$^+$ DCs after treatment with conditioned medium (= SN of 624-mel treated with T-VEC for 24 and 48h). As a negative control, cells were left untreated. Abbreviations: myDCs: myeloid dendritic cells ; SN: supernatant ; T-VEC: Talimogene laherparepvec ; UT: untreated.

Figure S9

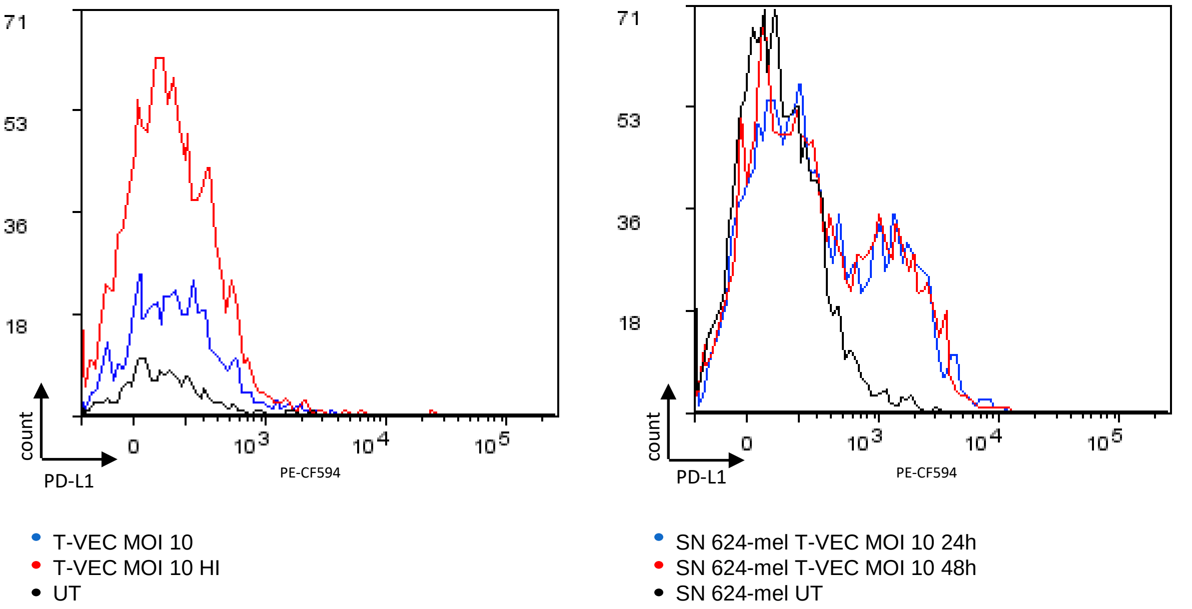

Figure S9: Representative histograms concerning the expression of PD-L1 on BDCA-1$^+$ myDCs after exposure to T-VEC, heat-inactivated T-VEC or conditioned medium. Histograms show the expression of PD-L1 on BDCA-1$^+$ DCs after treatment with active T-VEC, heat-inactivated T-VEC or conditioned medium (= SN of 624-mel treated with T-VEC for 24 and 48h). As a negative control, cells were left untreated. Abbreviations: HI: heat-inactivated; myDCs: myeloid dendritic cells ; MOI: multiplicity of infection ; PD-L1 : programmed death-ligand 1 ; SN: supernatant ; T-VEC: Talimogene laherparepvec ; UT: untreated.
